# Editorial: (p)ppGpp and Its Homologs: Enzymatic and Mechanistic Diversity Among the Microbes

**DOI:** 10.3389/fmicb.2021.658282

**Published:** 2021-03-25

**Authors:** Katarzyna Potrykus, Michael Cashel, Gert Bange

**Affiliations:** ^1^Department of Bacterial Molecular Genetics, Faculty of Biology, University of Gdańsk, Gdańsk, Poland; ^2^Intramural Program, Eunice Kennedy Shriver Institute of Child Health and Human Development, National Institutes of Health, Bethesda, MD, United States; ^3^Department of Chemistry, Center for Synthetic Microbiology (SYNMIKRO), Philipps-University Marburg, Marburg, Germany

**Keywords:** (p)ppGpp, stringent response, (p)ppApp, (p)ppGpp synthesis and degradation, enzymatic regulation, RSH, SAH, SAS

## Dr. Michael Cashel – The Role of Luck in the Discovery of (p)ppGpp

The background leading to the discovery of (p)ppGpp in *E. coli* began long ago when one of the goals in microbiology was to understand the biosynthetic pathways for DNA, RNA, and protein accumulation. Amino acid (AA) auxotrophs starved for one or more amino acid stopped growth and accumulation of all macromolecules, and this was referred to as the stringent response. A puzzle arose because mutants at a single locus (*relA*) in many strains continued to accumulate rRNA and tRNA for some time after growth had stopped due to AA starvation. The name for “RelA” is because the otherwise strong stringent RNA control in response to starvation was greatly relaxed in the mutant (Stent and Brenner, 1961). In addition, comparing *valS*^*ts*^ *relA* vs. *valS*^*ts*^ *relA*^+^ double mutants at restrictive temperatures suggested that limited charging of a single tRNA (valyl tRNA) can trigger the stringent response, despite the presence of an otherwise full array of charged tRNA (Neidhardt, 1966). Another puzzle was that adding chloramphenicol to non-growing AA starved cells could restore (relax) the rates of RNA accumulation (Kurland and Maaløe, 1962).

At about that time, Dr. Jon Gallant found that plasmolysis of cells in hypertonic 2M sucrose permeabilized them to actinomycin, which blocked incorporation of labeled NTP substrates by RNA polymerase (RNAP). Dr. Cashel's task for graduate training in the Gallant lab in Seattle was to ask if RNAP activity in plasmolyzed cells reflected the stringent/relaxed RNA control observed *in vivo*. The assay was to compare the RNA control response when labeling RNA with ^3^H-UTP vs. ^3^H-UMP and remaining cold ATP, GTP, and CTP rNTP substrates. The results suggested that all phosphotransfer might play an indirect role in the operation of the stringent response by inhibiting RNA polymerase through limiting the formation of UTP from UMP (Gallant and Cashel, 1967). Since uracil permeability was later shown to be severely inhibited by ppGpp, RNA synthesis estimates needed correction for uptake inhibition as well (Winslow and Lazzarini, 1969).

To verify the blocked phosphotransfer hypothesis, researchers asked whether phosphorylation of all ribonucleotides was similarly affected. This was probed in uniformly ^32^P labeled cells analyzed by two-dimensional PEI-cellulose thin layer chromatography (TLC) to visualize most nucleotides. This first step was development with Na-formate (pH 3.4), followed by a methanol wash, then developed in LiCl. Standard cell extraction was with strong acids. However, since formate was the first step, a shortcut for extraction was devised using Na-formate (pH 3.4) which allowed cell-free extracts to be spotted directly on chromatograms (Cashel and Gallant, 1969). This turned out to be an extremely lucky choice because (p)ppGpp was later found to be labile in strong acids, much more so than riboNTPs (Cashel and Kalbacher, 1971). The resulting TLC autoradiograms of ^32^Pi labeled, AA starved *relA*+ but not *rel*A- strains revealed different labeling patterns. One spot seemed to appear almost by magic, it increased in starved *relA*^+^ cells but disappeared for the starved *relA* mutant (Cashel and Gallant, 1969). Thus, it was called the “magic spot.” Later at NIH, Dr. Cashel devised a TLC method that in one dimension resolved this spot into two (MS I and MS II) and separated them from the GTP and GDP pool (Cashel et al., 1969). The two spots were later identified as ppGpp (MS I) and pppGpp (MS II). The initial work at NIH on (p)ppGpp benefited from advice from Drs. Bob Lazzarini and Maxine Singer.

This devised method was used for many years to study the kinetics of induction, reversal, and AA starvation specificity of the stringent response (Cashel and Gallant, 1969, Cashel and Kalbacher, 1971, Cashel et al., 1969). However, 50 years later this method was found to be inadequate because pppApp co-migrates with ppGpp, and ppApp comigrates with GDP (Sobala et al., 2019). The amounts of (p)ppApp in (p)ppGpp samples estimated by the old method now needed close scrutiny. One example described in this Research Topic comes from Dr. Jue D Wang's lab showing, by mass spectrometry, that products of the *B. subtilis* small alarmone synthetase SasA (synonyms: RelP, SAS2, YwaC) include pGpp, ppApp, and AppppA (Fung et al.).

Studies of (p)ppGpp by many researchers over the past half century in the microbial and plant kingdoms have revealed an astonishing regulatory diversity that was viewed in the beginning with healthy skepticism that dominated the early claims of global effects. This Research Topic hosted in Frontiers in Microbiology provides a glimpse of the enormous diversity of alarmones' actions emerging for (p)ppGpp, and possibly for (p)ppApp, and unquestionably for monocyclic, homocyclic, and heterocyclic nucleotides, barely mentioned here.

## Enzymatic and Mechanistic Diversity Among the Microbes–Manuscripts Accepted for This Research Topic

Over the years, protein structures supported by biochemical studies have led to identifying specific contact points between a variety of cellular proteins and/or ribosomes needed for synthesis and or degradation of an increasingly diverse array of (p)ppGpp and other potential regulatory nucleotides. While introduction of specific mutations/deletions has been exploited to discover a trove of biochemical effects, the conflicts among different studies are of special interest. In this Research Topic Takada et al. define specific mutants and deletions of the *Escherichia coli* RelA and *B. subtilis Rel* RRM domains. The authors conclude that deletion of the RRM domain, which yields uncontrollable (p)ppGpp production is not related to the loss of the enzyme's auto-inhibition, but instead is caused by misregulation of RelA/Rel by the ribosome. On the other hand, the Kaspy and Glaser manuscript is a follow up of their earlier work (Gropp et al., 2001) that seems to validate their conclusion that oxidation of the zinc-finger domain (localized next to RRM) induces inactive oligomers that are argued to regulate cellular RelA activity (Kaspy and Glaser). Each work has strong evidence that supports each authors' interpretations.

Since the initial discovery of RelA as a key enzyme of (p)ppGpp synthesis, a striking diversity of enzymes capable of synthesizing and degrading the alarmones has been unraveled. This is best illustrated by the class of small alarmone synthetases (SAS), which consist of only the (p)ppGpp synthetase domain. SAS enzymes are found in many bacteria including many relevant pathogens, such as *Staphylococcus aureus* (Steinchen and Bange, 2016).

In this Research Topic, Wolz et al. describe their latest findings on the functional roles of the SAS enzymes, RelP and RelQ, on the biofilm formation and maintenance under conditions of cell wall stress in *S. aureus* (Salzer et al.). Besides the canonical SAS enzymes, in some bacterial species such as *Mycobacterium smegmatis*, the dual domain SAS enzyme RelZ is found. In this “third” small alarmone synthetase, the (p)ppGpp synthetase domain is N-terminally extended by an RNAseHII domain. In their review, Krishnan and Chatterji discuss the functional roles of this class of SAS enzymes and conclude ways to translate the learned knowledge into ways combating persistent infections (Krishnan and Chatterji).

On the other hand, Wang et al. provide compelling evidence that the SAS enzyme RelP (also known as SasA or SAS1) is also able to produce the ppApp nucleotides, in addition to its original role as (p)ppGpp producer. Thus, it will be amazing to learn to which extent these enzymes are involved in the regulation of alarmones outside of (p)ppGpp, such as (p)ppApp and AppppA, which also appears to be affected by RelP (Fung et al.).

An example of diversity among enzymes hydrolyzing 3′-pyrophosphorylated nucleotides is presented by Potrykus et al.. The authors have developed a rapid real-time enzymatic assay which allows determining a given enzyme's ability to hydrolyze (p)ppGpp and (p)ppApp. Enzymes capable of hydrolyzing only (p)ppGpp (*Streptococcus equisimilis* RelSeq), only (p)ppApp (*Methylobacterium extorquens* SAH), and both (*Drosophila melanogaster* MESH1) were investigated. Although very intriguing, the functional consequences of such diversity are as of yet unknown.

Another study in this Research Topic on (p)ppGpp hydrolases is reported by Sanyal et al., who investigate (p)ppGpp degradation *in vivo* in the absence of SpoT and two Nudix enzymes—NudG and MutT—were found to participate in this process. Such an approach was possible through the use of *relA* hypomorphic mutants, as normally, deletion of *spoT* in the presence of intact *relA* is lethal. The conclusions are rather intricate and future *in vitro* studies with purified enzymes seem highly warranted to further support them.

Besides the diversity of enzymes involved in synthesis and degradation of (p)ppGpp, our knowledge on the molecular targets affected by the alarmones is steadily increasing. Thus far, over 30 different protein targets have been reported to be binding partners of (p)ppGpp. Thus, Kushwaha et al. set out to further detail how these chemically simple molecules achieve this binding diversity (Kushwaha et al.). Using molecular dynamics simulation studies, they show that phosphate chains provide molecular plasticity to (p)ppGpp nucleotides, enabling its binding diversity, while its guanosine-moiety might provide further specificity for certain target families, such as GTPases.

In addition, Steinchen et al. summarize all reported (p)ppGpp-binding targets (Steinchen et al.). When sorting and ranking them according to their known binding strengths, ppGpp and/or pppGpp show not only the enormous diversity of cellular processes affected, but much more suggests a “priority scheme” of targets modulated by the alarmones. Thus, (p)ppGpp appears to continuously modulate most of the microbial biochemistry rather than being part of an all-or-nothing switch between the “relaxed” and “stringent” states, as initially assumed in the beginning of their discovery.

A similar view is reflected in three articles addressing the importance of (p)ppGpp basal levels in cell physiology and adaptation. Spira and Ospino point out that there is great variability in (p)ppGpp basal levels in various *E. coli* laboratory strains and discuss the consequences this basal level might bring for the cell, such as its effect on bacterial pathogenicity, antimicrobial resistance, overall growth rate, and nutritional competence. Based on the evaluated data, the authors make an interesting conclusion that the *relA* and *spoT* genes are continuously undergoing a microevolutionary pressure so that the cells are producing (p)ppGpp basal levels that are optimal for a given population.

On the other hand, Imholz et al., noticed the problem of using different laboratory strains and techniques to evaluate the relationship between basal (p)ppGpp levels, growth rate control, and RNA synthesis in *E. coli*. In their perspective article, the authors re-evaluate some of the literature data and compare it with the results obtained by them with an LC-MS method. Although there was some variation, the inverse correlation between (p)ppGpp concentration and growth rate was preserved when growth was varied by nutritional conditions. The authors also performed experiments with strains where RNA polymerase (RNAP) (p)ppGpp binding sites were disrupted and found that disrupting one or the other site does not abolish this correlation. This may not be surprising since Myers et al. show by using Differential Radial Capillary Action of Ligand Assay (DRaCALA) assays that ppGpp has similar affinity to both of its binding sites on RNAP. Interestingly, upon binding of the first ppGpp molecule, binding of the second ppGpp molecule seems to be greatly enhanced, regardless of which site was occupied first. It is therefore intriguing whether the same would be true for pppGpp, as it was shown that in many instances ppGpp is a stronger effector than pppGpp (Mechold et al., 2013). Still, experiments with a double site mutant should be performed to provide a definitive answer to the interesting notion raised by Imholz et al. that the (p)ppGpp—RNA level inverse relationship might be controlled by some other factors than the binding of (p)ppGpp to RNAP.

The review by Fernandez-Coll and Cashel also deals with the importance of (p)ppGpp basal levels in the cell. The authors make a very important point of differentiating between (p)ppGpp acting as a second messenger (i.e., under “normal” growth, meaning a change in basal levels of (p)ppGpp), and as an alarmone (i.e., under stress conditions, when (p)ppGpp levels abruptly increase). Many researchers do not make this distinction and use those terms interchangeably while, in fact, they involve different cellular strategies for adaptation or survival. In addition, the authors point out that when considering (p)ppGpp metabolism as a potential antibiotic target, the focus should not be solely on its synthesis; perhaps its hydrolysis should be considered instead.

In light of the studies striving to develop novel antibiotics based on the notion that (p)ppGpp is known to be responsible for bacterial pathogenicity, it must be noted that recently Nowicki et al. (2019) have demonstrated that several isothiocyanates (ITC) cause *E. coli* growth inhibition by induction of the stringent response. This discovery is especially important for STEC strains (encoding Shiga toxins), since unlike antibiotics, ITCs do not induce Shiga toxin production. Here, the authors offer a follow-up on their previous findings and demonstrate that similar effects are observed when employing aliphatic ITCs, and what is even more important, some of these compounds act in a synergistic fashion (Nowicki et al.). Whether this means that their mechanism of action is different or not remains to be investigated.

In addition, this Research Topic offers three review articles focused on the role of (p)ppGpp in pathogenicity and adaptation to changing environmental conditions by bacteria other than *E. coli*. The article by Zhang et al., provides a comprehensive evaluation of (p)ppGpp's role in streptococci. In particular, (p)ppGpp synthesis, effects on physiology (including persistence and pathogenicity), transcriptional regulation, and a link between (p)ppGpp and CodY are discussed. On the other hand, the review by Das and Bharda, centers on description of (p)ppGpp metabolism in several different bacteria, with analysis of (p)ppGpp's role in production of antibiotics and in antibiotic resistance (several different mechanisms are described). Finally, Kundra et al. provide a very comprehensive and detailed review of (p)ppGpp's role in virulence of several Gram(+) and Gram(–) bacteria, as well as in *Mycobacteria* and *Borrelia burgdorferi*. The authors also provide a timely and thorough evaluation of targeting (p)ppGpp signaling by potential therapeutic agents (examples of compounds affecting its synthesis and hydrolysis are provided), as well as highlight the necessity of exploring the nature of crosstalk between (p)ppGpp and c-di-AMP.

Finally, the manuscript by Bartoli et al., is an example illustrating diversity among closely related enteric bacteria in regulation by the same transcriptional factors, in this case—SlyA. This factor was initially reported to be regulated by (p)ppGpp in *Salmonella enterica*, and thus the authors set out to use SlyA-regulated genes as reporters of ppGpp levels in *E. coli*. Although that attempt has failed and the authors disprove direct regulation of SlyA by (p)ppGpp, they make an important point that although SlyA may act through the same molecular mechanism in both bacteria, its physiological role in those bacteria is quite different.

## Author Contributions

KP, MC, and GB edited the Frontiers Research Topic on *(p)ppGpp and Its Homologs: Enzymatic and Mechanistic Diversity Among the Microbes* and wrote the editorial. All authors contributed to the article and approved the submitted version.

## Conflict of Interest

The authors declare that the research was conducted in the absence of any commercial or financial relationships that could be construed as a potential conflict of interest.
